# Sex Differences in Renal Outcomes and Metabolic Markers by Combination Therapy with SGLT2 Inhibitors and GLP-1 Receptor Agonists in Individuals with Type 2 Diabetes: A Post-Hoc Analysis of the RECAP Study

**DOI:** 10.31662/jmaj.2025-0224

**Published:** 2025-08-08

**Authors:** Yoshimi Muta, Kazuo Kobayashi, Masao Toyoda, Mari Sotozawa, Kyoji Chiba, Yuki Senda, Saki Hideshima, Yuichi Takashi, Hisashi Yokomizo, Takuya Hashimoto, Kei Takeshita, Shunichiro Tsukamoto, Miwako Yomota, Miwa Ota, Atsuhito Tone, Moritsugu Kimura, Takaya Matsushita, Daisuke Suzuki, Takashi Murata, Daisuke Tsuriya, Kouichi Tamura, Keizo Kanasaki, Daiji Kawanami

**Affiliations:** 1 Department of Endocrinology and Diabetes, Fukuoka University School of Medicine, Fukuoka, Japan; 2 Department of Medical Science and Cardiorenal Medicine, Yokohama City University Graduate School of Medicine, Kanagawa, Japan; 3 Division of Nephrology, Endocrinology and Metabolism, Department of Internal Medicine, Tokai University School of Medicine, Kanagawa, Japan; 4 Department of Nephrology, Yokohama Sakae Kyosai Hospital, Kanagawa, Japan; 5 Division of Endocrinology and Metabolism, 2nd Department of Internal Medicine, Hamamatsu University School of Medicine, Hamamatsu, Japan; 6 Department of Internal Medicine 1, Endocrinology and Metabolism, Shimane University Faculty of Medicine, The Center for Integrated Kidney Research and Advance, Faculty of Medicine, Shimane University, Izumo, Japan; 7 Department of Internal Medicine, Diabetes Center, Okayama Saiseikai General Hospital, Okayama, Japan; 8 Department of Diabetology, Endocrinology and Metabolism, Tokyo Medical University Hachioji Medical Center, Tokyo, Japan; 9 Suzuki Diabetes Clinic, Kanagawa, Japan; 10 Department of Clinical Nutrition, NHO Kyoto Medical Center, Kyoto, Japan; 11 Diabetes Center, NHO Kyoto Medical Center, Kyoto, Japan

**Keywords:** diabetic kidney disease, GLP-1 receptor agonists, sex differences, SGLT2 inhibitors, type 2 diabetes

## Abstract

**Introduction:**

We previously reported that combination therapy with SGLT2 inhibitors (SGLT2i) and GLP-1 receptor agonists (GLP-1Ra) was beneficial for the progression of diabetic kidney disease. However, sex differences in renal outcomes and metabolic markers with combination therapy remain unclear. To clarify this, we performed a post hoc analysis of the differences in renal outcomes between males and females.

**Methods:**

Among 643 individuals with type 2 diabetes (T2D) who had received their preceding medication for ≥6 months and concomitant medication for ≥12 months, data from 361 males and 282 females were analyzed in this post hoc study. Renal outcomes and changes in metabolic markers were analyzed. To adjust for confounding factors at baseline, a propensity score analysis with inverse probability weighting (PS-IPW) was adopted, and a generalized linear model was used for the comparison.

**Results:**

In the PS-IPW model, the incidence of renal composite outcomes was 28% in the male group and 26% in the female group, with an odds ratio of 0.90 (95% confidence interval: 0.56-1.44, p = 0.65). Significantly lower levels of diastolic blood pressure (DBP), mean arterial pressure, and alanine aminotransferase, and larger decreases in the body mass index and DBP were observed in the female group than in the male group.

**Conclusions:**

DBP reduction differed between males and females with T2D treated with a combination of SGLT2i and GLP-1Ra. Sex differences need to be considered clinically in combination therapy, and their impact on cardiovascular events should also be investigated in the future.

## Introduction

Diabetic kidney disease (DKD) is a major cause of end-stage kidney disease (ESKD). Risk factors of DKD consist of various factors such as hyperglycemia, hypertension, and dyslipidemia. Kidney metabolism under diabetic conditions may vary according to sex differences ^(1)^. However, sex differences in the incidence and progression of DKD are inconsistent in clinical studies ^(2)^. Some studies have reported that males with diabetes are at a higher risk of progressing to ESKD than females ^(3), (4)^. Experimental studies have suggested beneficial effects of estrogen and adverse effects of androgen in the kidneys ^(5), (6)^. However, clinical studies have failed to ameliorate DKD with estrogen replacement. Therefore, the significance of sex differences as therapeutic targets in DKD remains unclear. Furthermore, sex differences in metabolic markers that are associated with the pathogenesis of DKD have not been fully understood.

Recent studies have demonstrated that multidrug therapy centered on sodium-glucose cotransporter-2 inhibitors (SGLT2i) is effective in inhibiting the progression of DKD. We previously reported that combination therapy with SGLT2i and GLP-1 receptor agonists (GLP-1Ra) slowed the annual estimated glomerular filtration rate (eGFR) decline compared with monotherapy with SGLT2i or GLP-1Ra ^(7), (8)^. However, little is known about sex differences in the response to combination therapy with SGLT2i and GLP-1Ra in individuals with type 2 diabetes (T2D).

A recent study showed that sex was not associated with differences in the efficacy and major adverse cardiovascular event (MACE) reduction effects of SGLT2i, GLP1-Ra, and dipeptidyl peptidase 4 inhibitors ^(9)^. However, no study has yet investigated sex differences in renal outcomes following combination therapy with SGLT2i and GLP-1Ra. To this end, we performed a post hoc analysis of the differences in renal outcomes between males and females.

## Materials and Methods

### Study design

The design of the RECAP study has been reported previously ^(7)^. In brief, individuals with T2D who received both SGLT2i and GLP-1Ra between April 2010 and December 2021 and met the following criteria were eligible for inclusion in this study: received their preceding medication for ≥6 months, received concomitant medication for ≥12 months, and had clinical data from baseline, time of drug addition, and final observation available (Supplementary Figure S1).

The following data were collected: sex, age, height, body weight (BW) (kg), systolic blood pressure (SBP) (mmHg), diastolic blood pressure (DBP) (mmHg), mean arterial blood pressure (MAP), eGFR (mL/min/1.73 m^2^), glycated hemoglobin A_1c_ (HbA_1c_) (mmol/mol), urinalysis results as urine albumin-to-creatinine ratio (ACR) (mg/gCr) or qualitative proteinuria (g/gCr), alanine aminotransferase (ALT) (IU/l), aspartate aminotransferase (AST) (IU/l), platelet counts (×10^3^/μL), and concomitant medications (hypoglycemic drugs, antihypertensive drugs, and statins). The eGFR was determined as follows: eGFR (mL/min/1.73 m^2^) = 194 × age^-0.287^ × serum creatinine^-1.094^ × (0.739 for females) ^(10)^. Qualitative proteinuria values were converted to albuminuria values using the following formula: predicted ACR = exp (5.2659 + 0.2934 × log [min (PCR [protein-to-creatinine ratio]/50, 1)] + 1.5643 × log [max (min[PCR/500, 1], 0.1)] + 1.1109 × log [max (PCR/500, 1)] - 0.0773 × [if female] + 0.0797 × [if diabetic] + 0.1265 × [if hypertensive]) ^(11)^. Individuals with any of the following conditions were excluded from the study: type 1 diabetes, chronic dialysis, severe liver dysfunction (e.g., liver cirrhosis or severe infection), terminal-stage malignancy, pregnancy, or treatment discontinuation. Individuals who opted for participation during the study period were also excluded.

Based on the inclusion criteria, we extracted data from 643 individuals with T2D treated with SGLT2i and GLP-1Ra. In this post hoc analysis, the data of 361 males and 282 females were analyzed (Supplementary Figure S2).

Owing to the retrospective nature of the present study, the requirement for informed consent was waived, and an opt-out method was used to provide information about the study to patients via the Research Ethics Committee, Tokai University School of Medicine website. The present study was approved by the Institutional Review Board for Clinical Research of Tokai University, Japan, on December 6, 2021, and the approval code is “21R226”.

### Outcomes

The renal composite outcome was defined as the progression of albuminuria and/or a ≥30% decline in the eGFR. We also evaluated the changes in the eGFR and the logarithmic value of the ACR (LnACR).

### Missing value analyses

To account for missing data, we used the multiple imputation (MI) method. We replaced each missing value with a set of substituted plausible values by creating 100 complete filled-in datasets using MI with the chained-equation method.

### Propensity score analyses using inverse probability weighting

Propensity score (PS) analyses were conducted to minimize the influence of confounding factors. In the comparison between the male and female groups, in each dataset built using MI, the PS for the female group was calculated by a logistic analysis using the following covariates: age, sex, body mass index (BMI), SBP, DBP, HbA_1c_, eGFR, LnACR at baseline, AST, ALT, platelet count, FIB-4 index, history of T2D, use of concomitant medications at baseline, duration of treatment with the preceding drug, and combination therapy. Height and BW were markedly different between males and females, so these were not included in the calculation of PS for the female group. The inverse probability weighting (IPW) method using PS was used to analyze the outcomes. We selected the model using the stabilized average treatment effect weighting with trimming (individuals with PS <0.05 or PS >0.95 were excluded from further analyses) because this model showed the lowest standardized differences in covariates. Comparisons of renal outcomes and clinical characteristics after combination therapy were performed using a generalized linear model (GLM).

### Sensitivity analyses

We built a complete case analysis (CCA) set that consisted of individuals with all of the following data: age, sex, height, BW, blood pressure (BP) at office, eGFR, HbA_1c_, ACR, ALT, AST, platelet counts, and information on the concomitant treatment at baseline, addition, and final observation. According to these inclusion criteria, 239 individuals were excluded, and 404 individuals (229 males and 175 females) were analyzed for the CCA using the PS-IPW method with the same algorithm (Supplementary Figure S2). Multiple regression analysis was performed to identify the independent factors related to the change in BW before and after the combination treatment of SGLT2i and GLP-1Ra. A stepwise method was performed using covariates as follows: age, sex, BW, MAP, HbA_1_c, eGFR, LnACR, AST, ALT at baseline, history of T2D, use of concomitant medications at baseline, total observational periods, and the type of the preceding drug.

### Statistical analyses

The IBM SPSS Statistics software program (version 28.0; IBM Inc., Armonk, NY, USA) was used for all statistical analyses. Statistical significance was set at p < 0.05.

## Results

### Baseline characteristics

The baseline characteristics are presented in Table 1. In the unadjusted model in the left column, the male and female groups showed significant differences in BW (85.0 ± 19.4 kg vs. 72.3 ± 16.2 kg, p < 0.001), DBP (79.1 ± 13.2 mmHg vs.75.6 ± 12.4 mmHg, p < 0.001), ALT (40.5 ± 30.3 IU/mL vs. 34.0 ± 28.0 IU/mL, *p* = 0.01), use of pioglitazone (17% vs. 9%, p = 0.003), and use of β blockers (20% vs. 10%, p < 0.001) at baseline. In the PS-IPW model in the right column, the range of standardized differences in the covariates was 0.001-0.08, except for BW, which suggests a well-balanced model excluding BW. The total observation and combination treatment periods (months) were 61.1 ± 22.7 and 33.5 ± 16.8 in males, and 62.7 ± 25.6 and 34.2 ± 17.5 in females (Table 1).

**Table 1.** Clinical Baseline Characteristics in the Analysis for the Type of Preceding Drug.

	Unadjusted			PS-IPW model		
				(by stabilized ATE with trimming)		
	Males,	Females,	p-value^*^	Males,	Females,	Standardized difference
	n = 361	n = 282		n = 342^†^	n = 270^†^	
Age (years)	55.7 ± 12.5	56.6 ± 13.8	0.39	56.8 ± 13.4	56.7 ± 14.0	0.01
Type of preceding drug (SGLT2i-preceding) (%))	182 (50%)	130 (46%)	0.28	164 (48%)	126 (47%)	0.03
History of T2D > 10 years (%)	291 (81%)	227 (81%)	0.73	274 (80%)	217 (80%)	0.01
BW (kg)	85.0 ± 19.4	72.3 ± 16.2	< 0.001	83.8 ± 19.1	71.4 ± 14.8	0.84
BMI	29.5 ± 5.9	29.8 ± 6.1	0.50	29.4 ± 5.9	29.3 ± 5.5	0.02
SBP (mmHg)	133.1 ± 19.3	134.4 ± 17.8	0.37	133.9 ± 21.4	133.4 ± 17.2	0.03
DBP (mmHg)	79.1 ± 13.2	75.6 ± 12.4	< 0.001	77.7 ± 13.5	76.8 ± 12.4	0.07
MAP (mmHg)	97.1 ± 13.8	95.2 ± 12.3	0.07	96.4 ± 14.1	95.7 ± 12.4	0.06
HbA_1c_ (mmol/mol [%])	71.4 ± 18.0 (8.7 ± 1.6)	73.4 ± 18.0 (8.9 ± 1.6)	0.17	71.6 ± 19.6 (8.7 ± 1.8)	72.0 ± 16.7 (8.7 ± 1.5)	0.02
eGFR (mL/min/1.73 m^2^)	76.8 ± 27.6	80.7 ± 27.1	0.08	77.8 ± 29.0	78.9 ± 25.9	0.04
LnACR	3.87 ± 2.00	3.61 ± 1.82	0.09	3.74 ± 1.95	3.72 ± 1.79	0.01
AST (IU/mL)	29.7 ± 19.2	29.9 ± 20.9	0.91	29.2 ± 20.4	29.3 ± 20.1	0.01
ALT(IU/mL)	40.5 ± 30.3	34.0 ± 28.0	0.01	36.3 ± 26.7	36.0 ± 32.0	0.01
FIB-4 index	1.25 ± 0.70	1.33 ± 0.92	0.19	1.32 ± 0.87	1.29 ± 0.76	0.04
Duration of the preceding treatment (month)	27.6 ± 28.4	28.4 ± 20.2	0.59	28.2 ± 21.3	29.9 ± 21.8	0.08
Duration of the combination treatment (month)	33.5 ± 16.8	34.2 ± 17.5	0.59	33.9 ± 16.6	33.5 ± 18.3	0.02
Total duration of the study (month)	61.1 ± 22.7	62.7 ± 25.6	0.40	62.1 ± 24.2	63.4 ± 28.3	0.05
Type of SGLT2i
Ipragliflozin	33 (9%)	34 (12%)	0.15	35 (10%)	27 (10%)	0.01
Dapagliflozin	82 (23%)	76 (27%)	0.15	81 (24%)	62 (23%)	0.02
Tofogliflozin	38 (11%)	31 (11%)		37 (11%)	26 (10%)	0.04
Luseogliflozin	14 (4%)	18 (7%)		19 (6%)	12 (4%)	0.05
Canagliflozin	41 (11%)	26 (9%)		40 (12%)	31 (11%)	0.01
Empagliflozin	95 (26%)	52 (18%)		78 (23%)	71 (26%)	0.08
SGLT2i was changed during the treatment periods	58 (16%)	45 (16%)		52 (15%)	41 (15%)	0.001
Type of
GLP1-Ra
Liraglutide	133 (37%)	81 (29%)	0.29	115 (34%)	88 (33%)	0.02
Dulaglutide	134 (37%)	112 (40%)	0.29	131 (38%)	103 (38%)	0.01
Exenatide	4 (1%)	4 (1%)		5 (1%)	4 (1%)	0.002
Lixisenatide	6 (2%)	3 (1%)		5 (1%)	6 (3%)	0.06
GLP1-Ra was changed during the treatment periods	84 (23%)	82 (29%)		86 (25%)	69 (26%)	0.01
Concomitant medications						
Sulphonylureaa	119 (33%)	80 (28%)	0.21	109 (32%)	91 (34%)	0.04
Metformin	196 (54%)	163 (58%)	0.37	186 (54%)	143 (53%)	0.03
Insulin	150 (42%)	131 (47%)	0.21	147 (43%)	112 (41%)	0.03
Pioglitazone	61 (17%)	25 (9%)	0.003	40 (12%)	26 (10%)	0.07
αGI	55 (15%)	33 (12%)	0.20	43 (13%)	38 14%)	0.04
Glinide	16 (4%)	12 (4%)	0.91	13 (4%)	11 (4%)	0.01
RAS inhibitor	192 (43%)	134 (48%)	0.15	172 (50%)	135 (50%)	0.01
CCB	138 (38%)	100 (36%)	0.47	125 (37%)	91 (34%)	0.06
β blocker	73 (20%)	29 (10%)	< 0.001	55 (16%)	45 (17%)	0.02
MRB	13 (4%)	13 (5%)	0.52	16 (5%)	10 (4%)	0.05
Thiazide	28 (8%)	17 (6%)	0.39	22 (6%)	15 (6%)	0.04
Loop	22 (6%)	16 (6%)	0.82	20 (6%)	16 (6%)	0.01
Statin	184 (51%)	136 (48%)	0.49	168 (46%)	133 (49%)	0.01

Values are presented as the mean ± SD, n/total n (%), or median [lower quantile, upper quantile]. ^*^P-values by unpaired t-test or chi-square test. ^†^Calculated number of subjects after weighting. ATE: average treatment effect; αGI: alpha-glucosidase inhibitor; ALT: alanine aminotransferase; AST: aspartate aminotransferase; BMI: body mass index; BW: body weight; CCB: calcium channel blocker; DBP: diastolic blood pressure; eGFR: estimated glomerular filtration rate; FIB-4: fibrosis-4 index; GLP1-Ra: glucagon-like peptide-1 receptor agonist; HbA_1c_: glycated hemoglobin A_1c_; LnACR: natural logarithm of albumin-to-creatinine ratio; MAP: mean arterial pressure; MRB: mineralocorticoid receptor blocker; PS-IPW: propensity score-inverse probability weighting; RAS: renin-angiotensin system; SBP: systolic blood pressure; SGLT: sodium-glucose cotransporter; SGLT2i: sodium-glucose cotransporter 2 inhibitor; T2D: type 2 diabetes.

### Analyses of differences between males and females

Table 2 presents the results of the PS-IPW analysis based on the GLM. During the observation period, the incidence of renal composite outcomes was 28% in the male group and 26% in the female group, with an odds ratio (OR) of 0.90 (95% confidence interval [CI]: 0.56-1.44, p = 0.65). Values for DBP, MAP, and ALT were significantly lower in the female group than in the male group, with mean differences [95% CI] of −3.3 mmHg [−5.8 to −0.9] (*p* = 0.01), −2.8 mmHg [−5.1 to −0.5] (p = 0.02), and −3.9 IU/l [−7.4 to −0.4] (p = 0.03), respectively.

**Table 2.** Renal Outcomes and Clinical Characteristics after Combination Treatment in the Analysis for the Type of Preceding Drug.

	PS-IPW model by stabilized ATE with trimming		
	Males,	Females,	GLM^#^
	n = 342^＊^	n = 270^＊^	
Renal outcomes and function			
a) Incidence of renal composite outcome	96 (28%)	70 (26%)	0.90 [0.56-1.44], p = 0.65
Progression of ACR status	34 (19%)	44 (16%)	0.85 [0.50-1.45], p = 0.55
≥30% decrease in the eGFR	40 (12%)	30 (11%)	0.95 [0.49-1.86], p = 0.89
b) Changes in eGFR			
Annual changes in the eGFR (mL/min/1.73 m^2^/year)	−1.7 ± 3.9	−1.8 ± 3.9	−0.1 [−0.8 to 0.7], p = 0.89
c) Changes in LnACR	0.13 ± 1.55	−0.08 ± 1.62	−0.21 [0.06-0.31], p = 0.17
Clinical characteristics after combination treatment			
eGFR (mL/min/1.73 m^2^)	69.2 ± 26.9	71.6 ± 26.9	2.3 [−2.6 to 7.3], p = 0.35
LnACR	3.86 ± 1.90	3.64 ± 1.75	−0.23 [−0.55 to 0.09], p = 0.16
BW (kg)	79.6 ± 17.4	66.6 ± 14.7	−13.0 [−15.8 to −10.3], p < 0.001
BMI	27.9 ± 5.4	27.3 ± 5.5	−0.6 [−1.5 to 0.3], p = 0.21
SBP (mmHg)	129.2 ± 16.47	127.4 ± 16.1	−1.7 [−4.6 to 1.2], p = 0.25
DBP (mmHg)	75.7 ± 3.9	72.4 ± 11.8	−3.3 [−5.8 to −0.9], p = 0.01
MAP (mmHg)	93.5 ± 3.45	90.7 ± 11.5	−2.8 [−5.1 to −0.5], p = 0.02
HbA_1c_ (mmol/mol [%])	63.1 ± 16.1 (7.9 ± 1.5)	63.8 ± 15.9 (8.0 ± 1.5)	0.7 [−2.3 to 3.6] (0.1 [−0.2 to 0.3]), p = 0.67
AST (IU/L)	26.2 ± 5.0	24.8 ± 17.4	−1.4 [−4.4 to 1.7], p = 0.38
ALT (IU/L)	31.0 ± 22.5	27.1 ± 18.3	−3.9 [−7.4 to −0.4], p = 0.03
FIB-4 index	1.37 ± 0.75	1.41 ± 0.94	0.05 [−0.11 to 0.20], p = 0.56
Change in the clinical findings			
Change in BW (kg)	−4.2 ± 7.3	−4.8 ± 7.3	−0.6 [−1.8 to 0.6], p = 0.33
Change in BMI	−1.5 ± 2.5	−2.0 ± 2.9	−0.5 [−1.0 to −0.1], p = 0.03
Change in SBP (mmHg)	−4.7 ± 23.4	−5.9 ± 18.6	−1.2 [−5.0 to 2.6], p = 0.53
Change in DBP (mmHg)	−2.0 ± 13.8	−4.5 ± 12.9	−2.5 [−4.9 to −0.1], p = 0.04
Change in MAP (mmHg)	−2.9 ± 15.1	−5.0 ± 13.1	−2.1 [−4.6 to 0.4], p = 0.10
Change in HbA_1c_ (mmol/mol [%])	−8.5 ± 20.8 (−0.8 ± 1.9)	−8.2 ± 20.3 (−0.7 ± 1.9)	0.3 [−3.5 to 4.1] (0.0 [−0.3 to 0.4]), p = 0.89
Change in AST (IU/L)	−3.0 ± 19.8	−4.5 ± 19.5	−1.5 [−5.1 to 2.1], p = 0.42
Change in ALT (IU/L)	−5.3 ± 24.0	−8.9 ± 29.2	−3.6 [−8.9 to 1.7], p = 0.18
Change in FIB-4 index	0.05 ± 0.63	0.12 ± 0.74	0.07 [−0.06 to 0.21], p = 0.29

Values are presented as the mean ± SD, n/total n (%), or the difference [95% CI], and p-value. ^*^Calculated number of participants after weighting. ^＃^Data are presented as the OR for the female group compared to the male group, the mean difference [95% CI], and p-value analyzed by GLM. ALT: alanine aminotransferase; AST: aspartate aminotransferase; ATE: average treatment effect; BW: body weight; BMI: body mass index; CI: confidence interval; DBP: diastolic blood pressure; eGFR: estimated glomerular filtration; FIB-4 index: fibrosis-4 index; GLM: generalized linear model; HbA_1c_: glycated hemoglobin A_1c_; IPW: inverse probability weighting; LnACR: logarithmic value of urine albumin-to-creatinine ratio; MAP: mean arterial pressure; OR: odds ratio; PS: propensity score; SBP: systolic blood pressure.

We next investigated changes in each parameter and observed that the ΔDBP was significantly greater in females than in males (−2.5 mmHg, 95% CI: −4.9 to −0.1, p = 0.04), as was the ΔBMI (−0.5, 95% CI: −1.0 to −0.1, p = 0.03). The ΔSBP tended to be greater in the female group than in the male group, but no significant difference was observed.

### Sensitivity analysis findings

The results of the sensitivity analysis are presented in Supplementary Tables S1 and S2. After the PS-IPW method, the standardized differences in baseline characteristics, excluding BW, were all below 0.12, which suggests that this model was well balanced between the 2 groups.

There were no significant differences in renal outcomes or clinical parameters at the final observation between the 2 groups, except for the BW; however, based on the 95% CI values, lower levels of BMI and DBP and a larger decrease in BMI, DBP, and ALT were observed in the female group than in the male group, with mean differences [95% CI] of −1.0 [−2.3 to 0.3] (p = 0.12), −3.1 mmHg [−6.4 to 0.1] (p = 0.06), −0.6 [−1.4 to 0.1] (p = 0.10), −2.8 mmHg [−6.1 to 0.5] (p = 0.10), and −6.5 IU/L [−15.1 to 2.1] (p = 0.14), respectively. These results are consistent with those of the full analysis set (FAS) analysis.

## Discussion

In this study, we found that BP reduction was greater in females than in males with T2D following combination therapy with SGLT2i and GLP-1Ra.To our knowledge, the current study demonstrated, for the first time, the sex differences in BP reduction by the combination therapy of SGLT2i and GLP-1Ra.

A series of randomized controlled trials (RCTs) have demonstrated that both SGLT2i and GLP-1Ra have renoprotective effects in individuals with T2D. These studies have shown that both males and females have equal cardioprotective effects ^(12)^. Research on sex differences in DKD has been conducted with great interest ^(13)^. However, the results of clinical studies on sex differences in DKD are inconclusive ^(2)^. These findings may be due to age-related changes in sex hormones, menopausal status, and the duration of diabetes ^(12)^. However, sex differences in changes of metabolic markers by combination therapy with SGLT2i and GLP-1Ra have not been fully understood.

Recently, Hanlon et al. ^(9)^ investigated whether or not the efficacy of SGLT2i, GLP-1Ra, and DPP-4 inhibitors varied according to age and sex in individuals with T2D. Their network meta-analysis included 601 RCTs that were analyzed for HbA_1c_ reduction and MACE. The authors demonstrated that SGLT2i were more cardioprotective in older individuals than in younger individuals with T2D, despite a smaller reduction in HbA_1c_. In addition, GLP-1Ra was more cardioprotective in younger individuals with T2D than older individuals. There were no marked differences in efficacy according to sex, regardless of monotherapy or combination therapy, but renal outcomes were not analyzed in that study ^(9)^. The present study showed no marked differences in renal composite outcomes among individuals with T2D who were receiving SGLT2i and GLP-1Ra combination therapy in real clinical settings. However, no previous studies have reported sex differences in metabolic markers following combination therapy with SGLT2i and GLP-1Ra.

DBP is an independent risk factor for renal dysfunction and arteriosclerosis ^(14), (15)^. In the Hisayama study, it was reported that renal arteriosclerosis appears from a stage of prehypertension (SBP 120-139 mmHg or DBP 80-89 mmHg) and linearly increases with increasing BP ^(16)^. Therefore, it is important to decrease BP at an early stage to prevent DKD progression. The mechanisms by which greater DBP decreases are achieved in women than in men following combination therapy with SGLT2i and GLP-1Ra remain unclear. One possibility is that the larger ΔBMI decrease in females affected the DBP reduction. We observed a difference of -0.5 in females compared to males; this could cause a greater DBP reduction in females than in males. Our previous study demonstrated a significantly larger decrease in BW in the GLP1-Ra-preceding group compared to the SGLT2i-preceding group ^(7)^. The prevalence of the preceding drug was also balanced between males and females after PS-IPW in this post hoc study. We therefore consider that GLP-1Ra-preceding did not affect BW reduction. Multiple linear regression analysis using the CCA dataset demonstrated that GLP-1Ra-preceding, female, BW at baseline, age, the use of alpha glucosidase inhibitor, and mineralocorticoid receptor blocker were independent factors for change in BW (kg), with coefficient values [95% CI] were −1.76 [−3.06 to 0.45], p = 0.01, −2.17 [−3.58 to −0.76], p < 0.01, −0.13 [−0.18 to −0.09], p < 0.01, −0.07 [−0.13 to −0.02], p = 0.01, −2.75 [−4.59 to −0.91], p < 0.01, and −3.72 [−6.94 to −0.49], p = 0.02, respectively (Supplementary Table S3). We therefore speculate that males had an advantage in a decrease in BW because they had larger BW at baseline compared to females, despite the similar BMI. This might relate to the fact that there was no significant sex difference in the change in BW.

Another possibility is the involvement of differences in the exposure of GLP-1Ra between males and females. A previous exposure-response analysis of semaglutide demonstrated that BW loss increased linearly with increasing semaglutide exposure. Furthermore, BW loss was greater in females than in males, with a steeper slope for semaglutide exposure ^(17)^. Similar observations have been reported in a study using liraglutide. Wilding et al. ^(18)^ demonstrated that increased exposure to liraglutide was associated with greater BW loss in both males and females. They also found that females had greater BW loss than males, which could be attributed to the higher exposure in females than in males ^(18)^. Furthermore, they described a 32% higher exposure to liraglutide in females than in males of similar BW ^(18)^. However, no sex differences in the effects of SGLT2i on BW loss or BP reduction have been reported. Therefore, we speculated that the greater BP reduction in females than in males was caused by GLP-1Ra. A recent RCT reported that the combination of SGLT2i and GLP-1Ra showed a substantial and clinically significant reduction in 24-hour systolic BP compared with either treatment alone in individuals with T2D ^(19)^. Although there is no report that shows a direct relationship between GLP-1Ra use and a greater BP in females than in males, it is possible that higher exposure to GLP-1Ra, with a combination of SGLT2i, induced greater BP reduction in females than in males in our study.

Several limitations associated with the present study warrant mention. First, postmenopausal women were included in the study. Therefore, we cannot exclude the possibility that changes in sex hormones might have affected the results. Second, the exposure to GLP-1Ra was not measured in this study. Therefore, the precise mechanisms underlying the greater BP reduction in females than in males remain unclear. Third, sample size imbalance, with a relatively small number of female participants, may have limited the statistical power to detect sex differences in albuminuria. Based on the numerically larger decrease in urinary ACR (UACR) among females and the known variability of UACR measures, it is possible that a larger sample size could reveal a statistically significant sex difference in UACR reduction. Fourth, this study included individuals who have preserved kidney function, suggesting that non-DKD individuals were enrolled.

In conclusion, BP reduction differed between males and females with T2D treated with a combination of SGLT2i and GLP-1Ra. RCTs will be required to fully clarify sex differences in the combination therapy of SGLT2i and GLP-1Ra in individuals with T2D.

## Article Information

### Conflicts of Interest

Masao Toyoda received lecture fees from Boehringer Ingelheim, Eli Lilly, Novo Nordisk, Sumitomo, and Mitsubishi Tanabe and received subsidies from Super Light Water, TAKAGI, Roche DC, and LifeScan. Yuichi Takashi received lecture fees from Kyowa Kirin. Daisuke Tsuriya received lecture fees from Eli Lilly, Novo Nordisk, and Mitsubishi Tanabe. Atsuhito Tone received lecture fees from Eli Lilly Japan K.K., Sanofi K.K., Abbott Japan LLC, Terumo Corporation, and Dexcom Japan G.K. Kouichi Tamura has received honoraria/lecture fees from AstraZeneca, Novartis, Bayer, Otsuka Pharmaceutical, Boehringer Ingelheim, Fuji Pharma, Kyowa Kirin, Ono Pharmaceutical, Sanwa Kagaku, Mochida Pharmaceutical, Kowa, Eli Lilly, Novo Nordisk, commissioned clinical trials, contract research and joint research funding: AstraZeneca, Bayer, Novartis, Chinook, Otsuka Medical Devices, Novo Nordisk, Terumo, Variatris, and Kowa, and, Scholarship donations: Otsuka Pharmaceutical, Bayer, Mochida Pharmaceutical, and Boehringer Ingelheim. Keizo Kanasaki received lecture fees from Dainippon-Sumitomo Pharma, Astellas, Astra Zeneca, Ono, Otsuka, Taisho, Tanabe-Mitsubishi, Eli Lilly, Boehringer-Ingelheim, Novo Nordisk, Sanofi, Bayer, Novartis, and Kowa, and received research funding from Boehringer Ingelheim, Kowa, Mitsubishi Tanabe Pharma, and Bayer. Daiji Kawanami received consulting/lecture fees from Bayer Yakuhin Ltd., Mitsubishi Tanabe Pharma Corporation, Novo Nordisk Pharma Ltd., Sanofi K.K., and Sumitomo Pharma Co., Ltd.; and grants from Bayer Yakuhin Ltd., Nippon Boehringer Ingelheim Co., Ltd., Nipro Corporation, and Sumitomo Pharma Co., Ltd.

### Acknowledgement

We are grateful to all participants and acknowledge the support of the members of the RECAP study who contributed considerably to data collection.

### Author Contributions

All authors contributed to the study’s conception, design, and data collection. Analysis was performed by Kazuo Kobayashi, Mari Sotozawa, Kyoji Chiba, Yoshimi Muta, Daiji Kawanami, and Masao Toyoda. The draft of the manuscript was written by Yoshimi Muta, Kazuo Kobayashi, Masao Toyoda, and Daiji Kawanami. All authors contributed significantly, read and approved the final manuscript.

### Approval by Institutional Review Board (IRB)

The present study was approved by the Institutional Review Board for Clinical Research of Tokai University, Japan on December 6, 2021 and the approval code is “21R226.”

### Disclaimer

Kouichi Tamura is one of the Editors of JMA Journal and on the journal’s Editorial Staff. He was not involved in the editorial evaluation or decision to accept this article for publication at all.

## Supplement

Supplementary Materials
