# Supplementary material for: Sex Differences in Renal Outcomes and Metabolic Markers by Combination Therapy with SGLT2 Inhibitors and GLP-1 Receptor Agonists in Individuals with Type 2 Diabetes: A Post-Hoc Analysis of the RECAP Study
Source: JMA J. 2025 Aug 8;8(4):1269–75. doi: 10.31662/jmaj.2025-0224 (PMC12598152; doi:10.31662/jmaj.2025-0224)
Supplement: Supplementary Materials [file 2433-3298-8-4-1269-s001.pdf]

Figure Legends

Supplementary Figure S1. Schematic of the study participants.

Abbreviations: GLP-1Ra, glucagon-like peptide 1 receptor agonist. SGLT2i, sodium-glucose cotransporter inhibitor.

Supplementary Figure S2. Schematic of the study participants.

Abbreviations: GLP-1Ra, glucagon-like peptide 1 receptor agonist. SGLT2i, sodium-glucose cotransporter inhibitor.

Supplementary Table S1. Clinical baseline characteristics on the analysis for the type of preceding.

Values are presented as the mean±SD, n/total n (%), or median [lower quantile, upper quantile].

*P-values by unpaired t-test or chi-square test. †Calculated number of subjects after weighting. Abbreviations: αGI: alpha glucosidase inhibitor; ALT: alanine aminotransferase; AST: aspartate aminotransferase; ATE: average treatment effect; BMI: body mass index; BW: body mass index; CCB: calcium channel blocker; DBP: diastolic blood pressure; eGFR: estimated glomerular filtration; FIB-4 index: fibrosis-4 index; GLP-1Ra: glucagon-like peptide 1receptor agonists; HbA_{1c}: glycated hemoglobin A_{1c}; IPW: inverse probability weighting; LnACR: logarithmic value of urine albumin-to-creatinine ratio; MAP: mean arterial pressure; MRB: mineral corticoid receptor blocker; PS: propensity score; RAS: renin-angiotensin system; SBP: systolic blood pressure; SD: standard deviation; SGLT2: sodium-glucose cotransporter inhibitor; T2D: type 2 diabetes.

Supplementary Table S2. Renal outcomes and clinical characteristics after combination treatment in the analysis for the type of preceding drug.

Values are presented as the mean±SD, n/total n (%), or the difference [95% CI], and P-value.* Calculated number of participants after weighting. #Data are presented as the OR for the female group compared to the male group, the mean difference [95% CI], and p-value analyzed by GLM. Abbreviations: ALT: alanine aminotransferase; AST: aspartate aminotransferase; ATE: average treatment effect; BW: body weight; BMI: body mass index; CI: confidence interval; DBP: diastolic blood pressure; eGFR: estimated glomerular filtration; FIB-4 index: fibrosis-4 index; GLM: generalized linear model; HbA_{1c}: glycated hemoglobin A_{1c}; IPW: inverse probability weighting; LnACR: logarithmic value of urine albumin-to-creatinine ratio; MAP: mean arterial pressure; OR: odds ratio; PS: propensity score; SBP: systolic blood pressure.

Supplementary Table S3. Multiple regression linear analysis for change in BW.

Abbreviations: GLP-1Ra: GLP-1 receptor agonists; BW: body weight; αGI: alpha glucosidase inhibitor; MRB: mineral corticoid receptor blocker.

Supplementary Figure S1

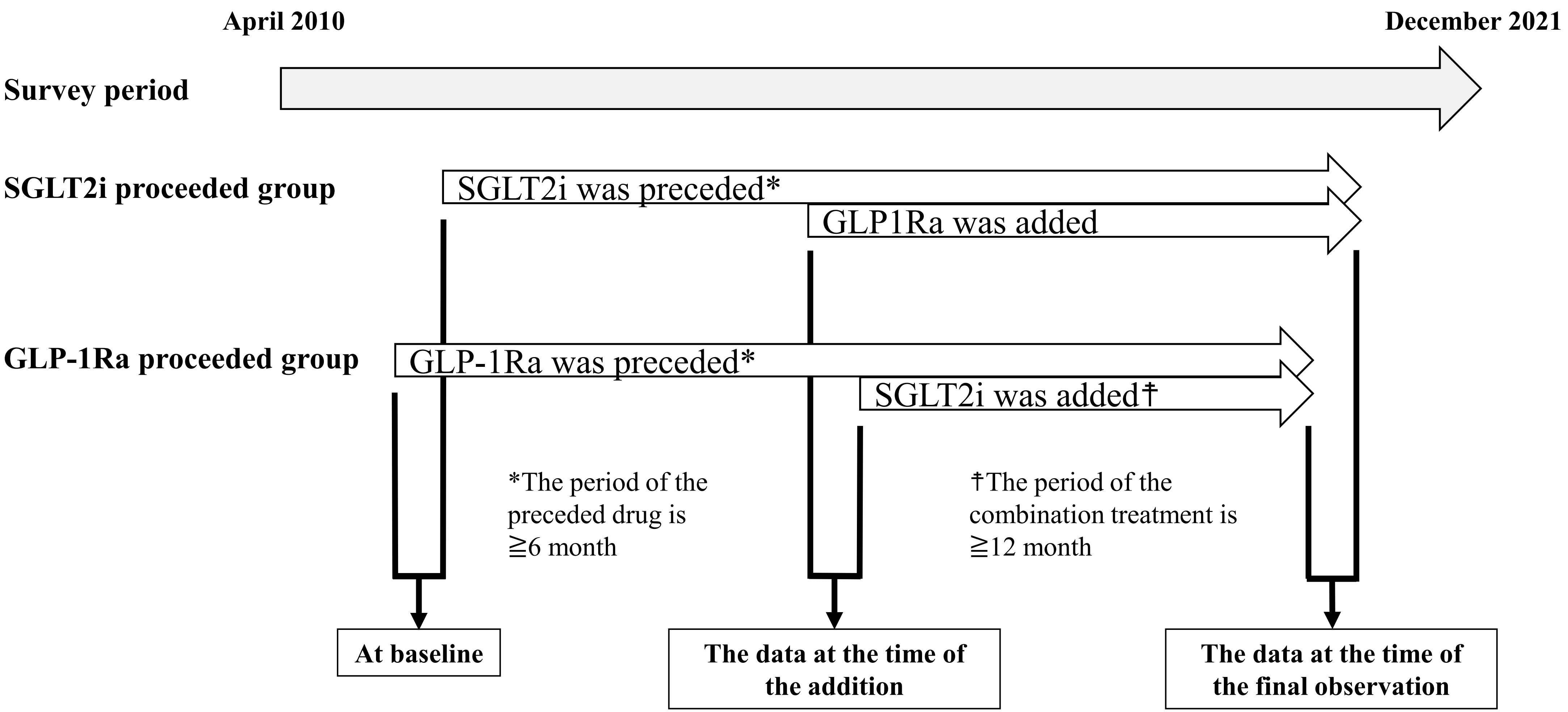

Supplementary Figure S2

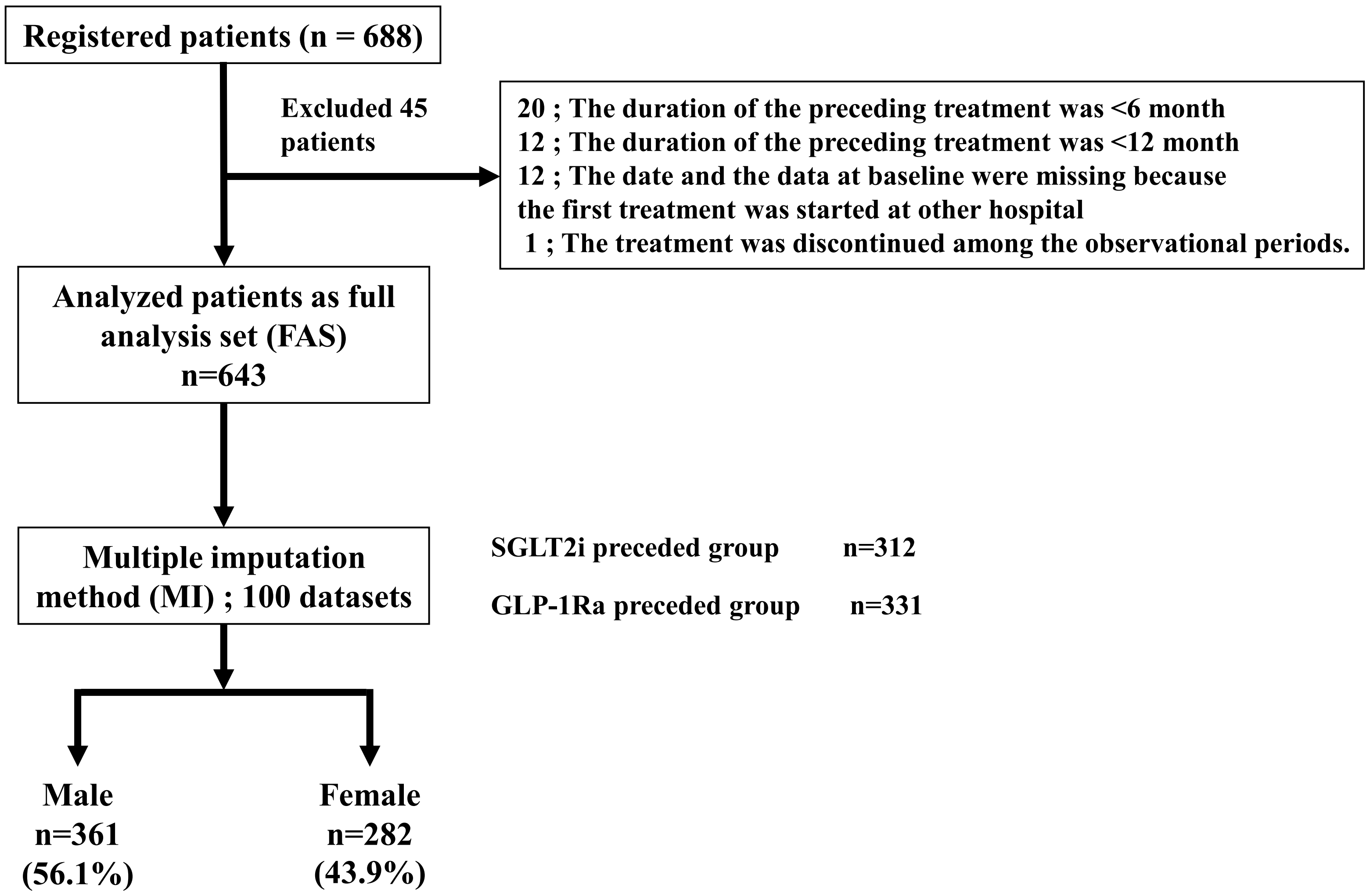

Supplementary Table S1

	Unadjusted			PS-IPW model (by stabilized ATE with trimming)		
	Males, N=229	Females, N=175	p-value*	Males, N=215†	Females, N=165†	Standardized difference
Age (years)	55.6±12.5	57.4±14.5	0.20	57.3±13.2	56.9±14.3	0.029
Type of preceding drug (SGLT2i-preceding) (%))	113 (49%)	73 (42%)	0.13	98 (46%)	73 (44%)	0.027
History of T2D >10 years (%)	180 (79%)	139 (79%)	0.84	162 (75%)	125 (75%)	0.010
BW (kg)	84.3±17.8	71.9±15.2	<0.001	84.4±18.4	69.9±14.0	0.872
BMI	29.2±5.4	29.9±5.6	0.20	29.4±5.6	29.0±5.0	0.075
SBP (mmHg)	134.3±19.3	134.8±17.5	0.79	135.0±18.9	134.9±17.3	0.005
DBP (mmHg)	80.0±13.2	75.3±11.9	<0.001	78.2±12.5	77.8±12.3	0.032
MAP (mmHg)	98.1±13.9	95.2±12.0	0.03	97.1±13.3	96.8±12.5	0.023
HbA_{1c} (mmol/mol [%])	71.1±18.2	73.5±18.3	0.18	71.2±18.7	72.5±17.0	0.072
eGFR (mL/min/1.73 m^2)	76.2±28.5	82.0±28.8	0.046	79.0±30.4	80.9±27.0	0.065
LnACR	4.0±1.9	3.7±1.6	0.06	3.74±1.74	3.67±1.54	0.045
AST (IU/mL)	29.7±19.7	28.2±17.8	0.44	28.2±17.3	29.3±17.9	0.063
ALT(IU/mL)	40.1±30.1	32.2±25.9	0.006	35.1±25.0	38.8±33.2	0.120
FIB-4 index	1.2±0.7	1.3±0.9	0.28	1.3±0.8	1.3±0.8	0.000
Duration of the preceding treatment (month)	27.4±18.3	28.4±21.3	0.60	27.3±18.4	30.0±24.4	0.127
Duration of the combination treatment (month)	33.8±16.4	35.1±18.6	0.48	34.8±15.7	34.7±18.5	0.006
Total duration of the study (month)	61.2±21.8	63.5±25.8	0.34	62.1±22.5	64.7±28.3	0.103
Type of SGLT2i						
Ipragliflozin	25 (11%)	19 (11%)	0.11	27 (13%)	21 (13%)	0.005
Dapagliflozin	38 (17%)	48 (27%)		41 (19%)	39 (23%)	0.112
Tofogliflozin	30 (13%)	27 (16%)		36 (17%)	21 (13%)	0.114
Luseogliflozin	10 (4%)	7 (4%)		8 (4%)	7 (3%)	0.027
Canagliflozin	24 (10%)	16 (9%)	0.11	23 (11%)	18 (11%)	0.007
Empagliflozin	70 (31%)	35 (20%)		52(24%)	42 (25%)	0.029
SGLT2i was changed during the treatment periods	32 (14%)	23 (13%)		26 (12%)	20 (12%)	0.001
Type of GLP1-Ra						
Liraglutide	96 (42%)	59 (34%)	0.53	85 (40%)	67 (40%)	0.022
Dulaglutide	70 (31%)	59 (34%)		69 (32%)	47 (29%)	0.079
Exenatide	3 (1%)	3 (2%)		5 (2%)	5 (3%)	0.044
Lixisenatide	5 (2%)	3 (2%)		3 (1%)	2 (1%)	0.016
GLP1-Ra was changed during the treatment periods	55 (24%)	51 (28%)	0.53	53 (25%)	44 (27%)	0.046
Concomitant medications						
Sulphonylureaa	76 (33%)	56 (32%)	0.80	69 (32%)	55 (33%)	0.026
Metformin	120 (52%)	98 (56%)	0.47	114 (53%)	84 (51%)	0.042
Insulin	99 (43%)	83 (47%)	0.40	92 (43%)	70 (42%)	0.007

Pioglitazone	39 (17%)	21 (12%)	0.16	24 (11%)	17 (10%)	0.028
αGI	42 (18%)	20 (11%)	0.06	28 (13%)	21 (13%)	0.009
Glinide	15 (7%)	8 (5%)	0.40	10 (5%)	8 (5%)	0.009
RAS inhibitor	126 (55%)	89 (51%)	0.41	111 (52%)	83 (50%)	0.027
CCB	93 (41%)	67 (38%)	0.64	84 (39%)	64 (39%)	0.006
β blocker	50 (22%)	17 (10%)	0.001	33 (15%)	29 (18%)	0.060
MRB	10 (4%)	7 (4%)	0.86	9 (4%)	6 (4%)	0.028
Thiazide	18 (8%)	12 (7%)	0.70	17 (8%)	10 (6%)	0.072
Loop	14 (6%)	7 (4%)	0.34	11 (6%)	8 (5%)	0.012
Statin	122 (53%)	83 (47%)	0.24	94 (44%)	77 (47%)	0.059

Supplementary Table S2

	PS-IPW model (by stabilized ATE with trimming)		
	Males, N=215*	Females, N=165*	GLM[#]
Renal outcomes and function			
a) Incidence of renal composite outcome	60 (28%)	37 (22%)	0.8 [0.4,1.3], p=0.33
Progression of ACR status	34 (16%)	21 (13%)	0.8 [0.4,1.5], p=0.48
Progression to microalbuminuria	20 (9%)	15 (9%)	1.0 [0.4,2.3], p=0.91
Progression to macroalbuminuria	14 (7%)	6 (4%)	0.6 [0.2,1.5], p=0.27
≥30% decrease in the eGFR	34 (16%)	20 (12%)	0.7 [0.3,1.5], p=0.38
b) Changes in eGFR			
Annual changes in the eGFR (mL/min/1.73 m^2/year)	-1.9±3.9	-1.9±3.8	0.0 [-1.0,1.1], p=0.94
c) Changes in LnACR	-0.1±1.3	-0.2±1.4	-0.1 [-0.5,1.9], p=0.41
Clinical characteristics after combination treatment			
eGFR (mL/min/1.73 m^2)	69.8±29.7	74.1±27.1	4.4 [-3.1,11.9], p=0.25
LnACR	3.7±1.8	3.5±1.5	-0.2 [-0.6,0.2], p=0.28
BW (kg)	80.3±17.0	65.0±14.7	-15.4 [-19.2, -11.6], p<0.001
BMI	28.0±5.1	27.0±5.5	-1.0 [-2.3,0.3], p=0.12
SBP (mmHg)	128.0±15.9	127.2±15.5	-0.9 [-4.7,2.9], p=0.65
DBP (mmHg)	75.8±13.1	72.7±11.4	-3.1 [-6.4,0.1], p=0.06
MAP (mmHg)	93.2±12.7	90.8±11.0	-2.4 [-5.5,0.7], p=0.13
HbA_{1c} (mmol/mol [%])	63.5±15.1	62.8±14.7	-0.7 [-4.1,2.6], p=0.67
AST (IU/L)	25.3±15.9	26.0±18.7	0.7 [-5.0,6.4], p=0.80
ALT (IU/L)	29.9±23.4	27.1±19.9	-2.8 [-7.5,1.9], p=0.24
FIB-4 index	1.3±0.7	1.5±0.9	0.2 [-0.1,0.4], p=0.22
Change in the clinical findings			
Change in BW (kg)	-4.1±6.2	-5.0±9.4	-0.9 [-3.0,1.2], p=0.41
Change in BMI	-1.4±2.1	-2.0±3.5	-0.6 [-1.4,0.1], p=0.10
Change in SBP (mmHg)	-7.0±21.6	-7.7±17.6	-0.7 [-5.7,4.3], p=0.78
Change in DBP (mmHg)	-2.4±13.8	-5.1±12.3	-2.8 [-6.1,0.5], p=0.10
Change in MAP (mmHg)	-3.9±14.9	-6.0±12.5	-2.1 [-5.6,1.4], p=0.24
Change in HbA_{1c} (mmol/mol [%])	-7.6±20.9	-9.7±19.3	-2.0 [-6.5,2.5], p=0.38
Change in AST (IU/L)	-2.8±18.8	-3.2±18.7	-0.4 [-5.5,4.8], p=0.89
Change in ALT (IU/L)	-5.2±23.3	-11.7±30.2	-6.5 [-15.1,2.1], p=0.14
Change in FIB-4 index	0.0±0.6	0.2±0.8	0.1 [-0.1,0.3], p=0.23

Supplementary Table S3

	coefficient value	95%CI [lower, upper]		p-value
GLP-1Ra -preceding	-1.76	-3.06	-0.45	0.01
female	-2.17	-3.58	-0.76	<0.01
BW at baseline	-0.13	-0.18	-0.09	<0.01
age at baseline	-0.07	-0.13	-0.02	0.01
use of αGI	-2.75	-4.59	-0.91	<0.01
use of MRB	-3.72	-6.94	-0.49	0.02
